# Health‐related quality of life of patients with multiple myeloma: A real‐world study in China

**DOI:** 10.1002/cam4.3391

**Published:** 2020-09-02

**Authors:** Xiaozhe Li, Junru Liu, Meilan Chen, Jingli Gu, Beihui Huang, Dong Zheng, Juan Li

**Affiliations:** ^1^ Department of Haematology The First Affiliated Hospital of Sun Yat‐sen University Guangzhou China

**Keywords:** health‐related quality of life, multiple myeloma

## Abstract

**Purpose:**

This study aimed to assess the health‐related quality of life (HRQOL) of Chinese patients with different stages of multiple myeloma (MM) who received various treatments and identify the factors associated with a lower quality of life in China.

**Methods:**

A cross‐sectional, anonymous questionnaire was distributed to adults with MM. The measures of quality of life included the European Organization for Research and Treatment of Cancer (EORTC) quality of life questionnaire (QLQ)‐C30, QLQ‐myeloma‐specific module 20 (MY20), and EuroQoL EQ‐5D. The data, including patient factors, difficulties experienced during the diagnosis and treatment processes, psychosocial factors and disease‐ or treatment‐related effects, were collected.

**Results:**

Four hundred and thirty patients with MM were recruited from all 27 provinces of China, and their average age was 55.7 years. Many variables were significantly associated with the HRQOL of the patients with MM. In the multivariate analyses, performance status, psychosocial factors, disease phase, and an early diagnosis were significantly associated with the HRQOL. In the subgroup analysis, the HRQOL of the patients who underwent autologous stem cell transplantation (ASCT) was significantly higher than that of the non‐ASCT patients. Treatment‐related toxicities had a significant impact on the quality of life of the patients with MM, and 91.5% of the patients intended to stop the maintenance treatment.

**Conclusions:**

The quality of life of patients with MM in China is affected by patient factors, difficulties experienced during the diagnosis and treatment processes, psychosocial factors, and disease‐ or treatment‐related effects. Efforts should be exerted to improve the overall quality of life of these patients in China.

## Graphical abstract

This study aimed to assess the health‐related quality of life (HRQOL) among Chinese patients with different stages of multiple myeloma (MM) who received various treatments and to determine which factors are associated with a lower quality of life in this population in China.

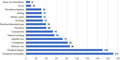

## 1 INTRODUCTION

Multiple myeloma (MM) is defined as the malignant proliferation of plasma cells and has an annual incidence of 3.29 to 4.82 per 100 000 individuals worldwide;^1^ MM remains a generally incurable disease. Patients often live with pronounced symptoms due to bone involvement and fractures, recurrent bacterial infections, impaired renal function, and anemia.^2^ Recently, improved survival rates have been reported in patients with MM due to the introduction of autologous stem cell transplantation (ASCT), alkylating agents, corticosteroids, proteasome inhibitors, immune modulators, and monoclonal antibodies.^3^ Although modern treatment approaches have positively influenced the overall survival (OS), the patients incur considerable costs. The burden of MM‐related symptoms, treatment‐related toxicities, and psychosocial effects adversely impact the health‐related quality of life (HRQOL). Patients with MM report a lower HRQOL than patients with other hematological malignancies. Thus, the optimization of the HRQOL of patients with MM is an important treatment goal.^4^, ^5^

Published studies provide detailed descriptions of the quality of life of patients with MM; however, these studies were primarily conducted in Western populations.^6^, ^7^, ^8^ To the best of our knowledge, no studies of HRQOL in Chinese patients with MM have been conducted. The quality of life may vary according to the cultural and health service contexts. In China, patients with MM experience social, economic, and emotional difficulties during the diagnosis and treatment processes, including the absence of a caregiver, a low family budget, long distance between the home and the hospital, and difficulties in early diagnosis. Because of these difficulties, an evaluation of the HRQOL of Chinese patients with MM is important to suggest strategies to improve HRQOL. Moreover, most studies examining the HRQOL of patients with MM are based on data from clinical trials.^9^, ^10^ The patients included in clinical trials are not representative of the general MM population, as clinical trial participants are often younger, and have a better performance status.^11^ Therefore, population‐based HRQOL studies including elderly and frail patients with MM are needed.

Here, we conduct a cross‐sectional survey of HRQOL data from a large number of patients with distinct stages of MM receiving various treatments in China. The goals of this study were to determine which factors were associated with a lower HRQOL and strategies to improve the HRQOL.

## 2 METHODS

### 2.1 Participants

For this cross‐sectional study, patients with MM were recruited from China. The survey was made available as a link on the website. The inclusion criterion for this study was a confirmed diagnosis of MM. Printed copies of the survey were also distributed at patient education conferences, advocacy meetings, and the inpatient or outpatient hematology clinics at The First Affiliated Hospital of Sun Yat‐sen University.

### 2.2 Survey

The survey was conducted from September 2017 to April 2018. The anonymous multiple‐choice questionnaire consisted of two dimensions. The first dimension included 30 questions assessing patient factors (gender, age, marital status, occupation status, education level, comorbidities, household registration, and performance status), difficulties experienced during the diagnosis and treatment processes (yearly family income, caregivers, interval from symptom onset to diagnosis, and distance between the home and the hospital), psychosocial factors and disease‐ or treatment‐related effects (disease phase, ASCT, and treatment‐related toxicities). The second dimension was HRQOL, which was measured by the European Organization for Research and Treatment of Cancer (EORTC) Quality of Life Questionnaire QLQ‐C30^12^ with the myeloma‐specific module (MY20)^13^ and the EuroQoL 5D‐3L questionnaire (EQ‐5D).^14^ These questionnaires have been validated in China and were used in our study. Performance status was assessed by the Eastern Cooperative Oncology Group (ECOG) scale.^15^ The presence of psychosocial factors was measured using the Hospital Anxiety and Depression Scale (HADS).^16^

QLQ‐C30, QLQ‐MY20, and EQ‐5D domains were scored in accordance with their published guidelines. The results were transformed into scales ranging from 0 to 100 points for the QLQ‐MY20 and QLQ‐C30. The QLQ‐C30 is a validated 30‐item questionnaire that incorporates nine multi‐item scales: five functional scales (physical, role, cognitive, emotional, and social functioning), three symptom scales (fatigue, pain, and nausea/vomiting), and a global health status/quality of life (QoL) scale. Six single‐item scales are also included (dyspnea, insomnia, loss of appetite, constipation, diarrhea, and financial difficulties). The QLQ‐MY20 is a supplemental MM‐specific 20‐item module. It includes two functional domains (future perspective and body image) and two symptom domains (disease symptoms and side effects of treatment). For the functional scales, higher scores indicate better HRQOL, whereas for the symptom scales, lower scores indicate a better health state. Health utility values were derived from the EQ‐5D using the UK general population weights algorithm, which provides a range of scores from the worst imaginable health state (−0.594) to the best imaginable health state (1.000).

The HADS is a 14‐item scale, with seven items each assessing depression or anxiety and two subscale scores ranging from 0 to 21 points. A cut‐off score of 8 of 21 points on each subscale was used to define clinical cases of depression or anxiety, and higher scores indicated higher levels of depression or anxiety.^17^ The current disease phase of MM was classified as unstable (at diagnosis, during periods of active treatment, and when experiencing progressive disease) or stable (treatment‐free interval).

### 2.3 Statistical analysis

All tests were two‐sided and considered statistically significant if *P* < .05 and were performed using IBM SPSS (version 22.0). The results of the descriptive analysis are presented as the means ± standard deviations (SD) or numbers (percentages), as appropriate. Pearson's Chi‐squared (for categorical variables) and t tests (for continuous variables) were used to measure between‐group differences in variables. Data regarding the patient factors, difficulties experienced during the diagnosis and treatment processes, psychosocial factors, and disease‐ or treatment‐related effects hypothesized to be associated with the patients’ HRQOL were obtained. To evaluate the association between these characteristics and the HRQOL, parametric P‐values were obtained via univariate subgroup comparisons using a t‐test to compare the scores between two groups. Multivariate linear regressions were also performed to determine the independent contributions of these factors to identify the key drivers of the overall HRQOL. The global quality of life scale of the QLQ‐C30 and the EQ‐5D index were the dependent variables, and the clinical, treatment, and demographic variables were the independent variables. Regarding the construction of the multivariate models, the variables found to be significant in the univariate analyses of all subjects, including sociodemographic, disease, and treatment history variables, and HADS depression and anxiety scores, were entered into the multivariate model, which was further reduced by excluding the nonsignificant factors.

## 3 RESULTS

A total of 451 questionnaires were collected, including 268 (59%) from the internet respondents and 183 (41%) from hard copy respondents at patient advocacy meetings, education conferences, and the inpatient or outpatient clinic. Patients resided in all 27 provinces of China. Twenty‐one patients who did not complete the entire survey were excluded. Data from 430 valid questionnaires are included in this report.

### 3.1 Patient factors associated with HRQOL

Table 1 displays the sample characteristics of 430 patients with MM. A general trend toward better EORTC QLQ‐C30 plus QLQ‐MY20 scores and EQ‐5D scores at baseline was observed among patients with the following characteristics: younger age (<65 years), male gender, working status, higher education level (at least a bachelor's degree), urban residency, and an ECOG 0‐1 degree (see Table 2).

**Table 1:** Characteristics of the subjects

Variable	Patients	
	n	%
a) Patient factors		
Gender	55.7 (SD 10.7, range 21‐91)	
Male	237	55.1
Female	193	44.9
Marital status		
Single	11	2.6
Married	398	92.6
Divorced	12	2.8
Widowed	9	2.1
Occupational status		
Working	97	22.6
Not working	333	77.4
Education level		
Less than secondary school	359	83.5
Bachelor's or higher degree	71	16.5
Comorbidities		
0	192	44.7
1	139	32.3
>2	99	23.0
Household registration		
Urban	342	79.5
Rural	88	20.5
ECOG		
0	43	10.0
1	234	54.4
2	94	21.9
3	48	11.2
4	11	2.6
b) Difficulties experienced during diagnosis and treatment processes		
Yearly family income		
>30 000 RMB	304	70.7
<30 000 RMB	126	29.3
Assistance from caregivers		
Yes	265	61.6
No	165	38.4
Interval from symptom to diagnosis		
<1 month	72	16.7
>1 month	358	83.3
Distance between the home and the hospital		
In the same city	188	43.7
In a different city	242	56.3
c) Psychosocial factors		
Depression		
Yes	191	44.4
No	239	55.6
Anxiety		
Yes	204	47.4
No	226	52.6
d) Disease‐ or treatment‐related effects		
Date of diagnosis		
< 12 m	198	46.0
12‐24 m	64	14.9
24‐36 m	57	13.3
36‐48 m	41	9.5
48‐60 m	24	5.6
>60 m	46	10.7
Disease phase		
Stable	248	57.7
Unstable	182	42.3
Current MM treatment		
Bortezomib	103	24.0
Lenalidomide	87	20.2
Thalidomide	77	17.9
Interferon	11	2.6
Bortezomib + lenalidomide/thalidomide	21	4.9
Not clear or no drug	131	30.5
ASCT		
Yes	118	48.8
No	124	51.2
Treatment‐related toxicities	3.16 (SD 1.65, range 0‐6)	
≤3	173	57.9
>3	126	42.1

**Table 2:** Univariate comparison of the EORTC QLQ‐C30 plus QLQ‐MY20 subscales and EQ‐5D scores of patients grouped by baseline characteristics

		QLQ‐C30 function domain scores					QLQ‐C30 symptom domain scores				
	Global health status	Cognitive functioning	Emotional functioning	Physical functioning	Role functioning	Social functioning	Appetite loss	Constipation	Diarrhea	Dyspnea	Fatigue
Gender											
Male (237)	60.51 (25.43)	77.78 (20.94)	74.12 (21.59)	72.49 (24.00)	73.14 (30.15)	55.20 (29.65)	20.39 (27.30)	21.52 (25.33)	10.13 (19.18)	21.52 (23.19)	38.02 (23.66)
Female (193)	55.61 (25.43)	73.06 (22.55)	68.61 (24.06)	69.12 (23.34)	69.52 (31.72)	52.25 (28.53)	24.01 (28.55)	24.18 (29.11)	11.74 (20.42)	23.32 (25.97)	43.87 (24.26)
*P* value	**.043**	**.025**	**.013**	.143	.227	.296	.182	.312	.399	.449	**.012**
Age											
<= 65 y (357)	59.45 (24.54)	76.42 (20.84)	72.01 (22.94)	72.55 (22.82)	72.61 (30.44)	53.69 (28.59)	20.17 (27.44)	21.48 (26.67)	10.36 (19.55)	20.92 (23.80)	39.78 (23.42)
> 65 y (73)	52.74 (26.57)	71.92 (25.74)	69.86 (22.59)	63.29 (26.65)	66.21 (32.63)	54.79 (31.97)	31.05 (28.51)	28.77 (28.50)	13.24 (20.59)	29.22 (26.61)	44.90 (26.80)
*P* value	**.036**	.107	.465	**.002**	.108	.768	**.002**	**.036**	.257	**.008**	.098
Marital status											
Married (398)	58.48 (25.23)	75.84 (21.66)	71.86 (22.69)	71.27 (23.22)	71.94 (30.16)	54.23 (28.97)	22.19 (28.02)	22.70 (27.21)	10.64 (19.69)	22.19 (24.39)	40.73 (23.98)
Single (32)	56.25 (22.10)	73.44 (23.52)	69.01 (25.24)	67.29 (29.59)	66.15 (38.91)	49.48 (31.53)	19.79 (26.59)	22.92 (26.01)	13.54 (20.49)	23.96 (25.73)	39.58 (25.70)
*P* value	.628	.549	.498	.362	.307	.376	.640	.965	.424	.695	.796
Occupational status											
Employed (97)	65.21 (23.23)	80.58 (19.64)	74.66 (20.62)	79.86 (20.48)	77.15 (28.39)	60.82 (26.25)	16.15 (26.40)	20.27 (24.79)	10.31 (18.86)	16.84 (21.04)	34.02 (22.04)
Unemployed (333)	56.31 (25.16)	74.22 (20.62)	70.77 (23.44)	68.39 (24.02)	69.87 (31.42)	51.85 (29.68)	23.72 (28.12)	23.42 (27.72)	11.01 (20.01)	23.92 (25.18)	42.58 (24.34)
*P* value	**.002**	**.011**	.141	**<.001**	**.041**	**.007**	**.018**	.314	.758	**.012**	**.002**
Education level											
<Bachelor's degree (359)	56.10 (24.90)	74.14 (22.30)	70.52 (22.89)	69.43 (24.03)	70.29 (31.55)	53.34 (29.45)	23.77 (28.59)	25.07 (27.91)	11.79 (20.55)	23.86 (25.10)	41.91 (23.89)
>= Bachelor's degree (71)	69.48 (22.49)	83.33 (17.14)	77.35 (22.06)	78.78 (20.63)	77.70 (26.57)	56.57 (27.67)	13.15 (22.17)	10.80 (18.49)	6.10 (14.15)	14.55 (19.30)	34.27 (24.18)
*P* value	**<.001**	**.001**	**.021**	**.002**	.065	.394	**.003**	**<.001**	**.026**	**.003**	**.014**
Household registration											
Urban (342)	59.06 (24.72)	76.61 (20.94)	73.42 (21.74)	72.20 (23.04)	72.47 (30.01)	56.09 (28.5)	21.64 (27.72)	22.51 (27.43)	10.72 (19.49)	21.73 (24.59)	39.96 (23.61)
Rural (88)	55.4 (25.96)	71.97 (24.57)	64.77 (25.82)	66.21 (25.87)	67.80 (33.98)	45.27 (30.21)	23.48 (28.66)	23.48 (25.85)	11.36 (20.77)	24.62 (23.97)	43.31 (25.77)
*P* value	0.220	0.075	**0.001**	**0.035**	0.207	**0.002**	0.580	0.765	0.786	0.324	0.245
ECOG											
0‐1 degrees (277)	65.31 (22.41)	80.75 (18.01)	77.35 (20.04)	82.26 (12.25)	83.75 (19.58)	63.18 (26.12)	13.72 (20.76)	18.65 (24.92)	10.11 (19.51)	16.85 (19.99)	31.13 (18.07)
2‐4 degrees (153)	45.64 (24.5)	66.45 (24.85)	61.33 (24.10)	50.54 (25.79)	49.35 (35.04)	37.04 (26.78)	37.04 (32.57)	30.07 (29.31)	12.20 (20.14)	32.24 (28.46)	57.88 (24.07)
*P* value	**<.001**	**<.001**	**<.001**	**<.001**	**<.001**	**<.001**	**<.001**	**<.001**	.293	**<.001**	**<.001**

	QLQ‐C30 symptom domain scores				QLQ‐MY20				EQ‐5D	
	Insomnia	Nausea and vomiting	Pain	Financial difficulty	Disease symptoms	Side effects of treatment	Body image	Future perspective	EQ‐5D index score	EQ‐5D VAS
Gender										
Male	24.33 (28.18)	8.65 (16.92)	29.75 (27.06)	52.88 (35.22)	22.78 (20.33)	24.75 (15.26)	69.06 (26.38)	61.79 (22.46)	0.68 (0.19)	67.43 (20.66)
Female	33.51 (30.90)	10.79 (19.47)	31.52 (26.21)	56.82 (34.54)	24.55 (19.72)	30.76 (16.68)	58.55 (29.62)	54.86 (24.68)	0.67 (0.19)	63.72 (22.16)
*P* value	**.001**	.223	.493	.245	.364	**<.001**	**<.001**	**.002**	.535	.073
Age										
<= 65 y (357)	28.48 (30.42)	9.15 (17.76)	29.51 (26.72)	56.77 (34.46)	23.26 (20.37)	27.28 (15.82)	63.31 (28.07)	58.42 (23.84)	0.69 (0.18)	66.75 (21.26)
> 65 y (73)	28.31 (26.45)	11.87 (19.74)	35.62 (25.69)	44.29 (35.60)	25.11 (18.50)	28.26 (17.93)	69.41 (29.27)	59.97 (23.18)	0.64 (0.22)	60.93 (21.62)
*P* value	.965	.243	.074	**.005**	.473	.637	.094	.611	**.037**	**.034**
Marital status										
Married (398)	28.89 (29.92)	9.72 (18.15)	30.28 (26.29)	54.44 (35.03)	23.53 (20.00)	27.12 (15.91)	64.74 (28.08)	58.63 (23.74)	0.68 (0.19)	65.72 (21.38)
Single (32)	22.92 (27.35)	8.33 (17.96)	22.85 (31.25)	57.29 (34.11)	24.13 (21.05)	31.56 (18.97)	59.38 (31.38)	59.38 (23.71)	0.66 (0.22)	66.28 (22.01)
*P* value	.275	.679	.466	.657	.871	.135	.303	.864	0.490	0.887
Occupational status										
Employed (97)	19.93 (27.91)	8.59 (16.34)	24.05 (26.46)	47.77 (33.65)	20.27 (20.41)	23.30 (14.97)	68.73 (25.83)	62.31 (23.22)	0.73 (0.14)	70.00 (19.47)
Unemployed (333)	30.93 (29.85)	9.91 (18.62)	32.43 (26.46)	56.66 (35.09)	24.54 (19.87)	28.69 (16.32)	63.06 (28.93)	57.62 (23.78)	0.66 (0.20)	64.53 (21.81)
*P* value	**.001**	.529	**.006**	**.027**	.065	**.003**	.083	.086	**.002**	**.027**
Education level										
<Bachelor's degree (359)	29.25 (29.93)	10.45 (18.69)	31.94 (26.94)	56.82 (34.64)	24.70 (20.14)	28.35 (16.23)	64.62 (28.11)	58.03 (22.84)	0.67 (0.20)	64.00 (21.86)
>= Bachelor's degree (71)	24.41 (28.71)	5.40 (14.30)	23.47 (24.17)	43.66 (34.55)	17.92 (18.76)	23.38 (15.38)	62.91 (29.57)	61.97 (27.64)	0.74 (0.14)	74.66 (16.35)
*P* value	.211	**.032**	**.014**	**.004**	**.009**	**.020**	.642	.201	**.004**	**<.001**
Household registration										
Urban (342)	28.36 (29.52)	8.72 (16.58)	29.19 (26.5)	50.88 (34.21)	22.81 (19.71)	26.46 (15.41)	64.33 (27.63)	60.88 (23.48)	0.68 (0.18)	66.65 (21.42)
Rural (88)	28.79 (30.82)	13.07 (22.95)	35.8 (26.81)	69.32 (33.99)	26.58 (21.2)	31.29 (18.47)	64.39 (31.07)	50.13 (22.73)	0.65 (0.21)	62.30 (21.12)
*P* value	.905	.098	**.038**	**<.001**	.116	**.012**	.984	**<.001**	.178	.088
ECOG										
0‐1 degrees (277)	21.9 (26.8)	4.75 (10.54)	20.88 (19.04)	48.13 (34.07)	17.19 (15.55)	23.09 (14.07)	70.52 (24.92)	63.62 (21.91)	0.75 (0.11)	71.89 (18.58)
2‐4 degrees (153)	40.31 (31.22)	18.41 (24.57)	48.04 (29.49)	66.45 (33.44)	35.15 (22.06)	35.34 (16.78)	53.16 (30.69)	49.75 (24.29)	0.54 (0.22)	54.67 (21.78)
*P* value	**<.001**	**<.001**	**<.001**	**<.001**	**<.001**	**<.001**	**<.001**	**<.001**	**<.001**	**<.001**

Bold values denote significant *P* values (<.05).

### 3.2 Associations between difficulties experienced during the diagnosis and treatment processes with HRQOL

The questionnaire mainly assessed four aspects of difficulties experienced during the diagnosis and treatment processes: yearly family income, caregivers, the interval from symptom onset to diagnosis, and the distance between the home and the hospital. Of the 430 respondents, 95 (22.1%) patients had a yearly family income level of > 30 000 RMB, 165 (38.4%) patients indicated an absence of caregivers, 358 (83.3%) respondents did not receive an early diagnosis of myeloma (less than 1 month), and 188 (43.7%) respondents were treated in the same city as their places of residence (see Table 1). In the analysis of HRQOL, patients with caregivers and a higher yearly family income (>30 000 RMB) received better scores on the functioning and symptom subscales and global health status of QLQ‐C30 plus QLQ‐MY20 and EQ‐5D index (see Table 3). In almost all domains, patients who reported a longer interval from symptom onset to diagnosis (>1 month) showed significantly lower levels of HRQOL. The location of the hospital in the same city as their home only produced differences in HRQOL in the cognitive functioning domain and symptom domain.

**Table 3:** Univariate analysis of the EORTC QLQ‐C30 plus QLQ‐MY20 subscales and EQ‐5D scores of patients grouped according to difficulties experienced during the diagnosis and treatment processes, psychosocial, factors and disease‐ or treatment‐related effects

		QLQ‐C30 function domain scores						QLQ‐C30 symptom domain scores			
	Global health status	Cognitive functioning	Emotional functioning	Physical functioning	Role functioning	Social functioning	Appetite loss	Constipation	Diarrhea	Dyspnea	Fatigue
Difficulties experienced during the diagnosis and treatment processes											
With caregivers											
Yes (265)	55.28 (24.83)	75.60 (22.520)	70.72 (23.15)	66.42 (25.710)	66.98 (32.35)	52.14 (29.11)	25.66 (29.66)	23.52 (27.60)	10.69 (19.65)	23.14 (25.48)	43.61 (24.94)
No (165)	63.18 (24.56)	75.76 (20.62)	73.13 (22.40)	78.30 (17.940)	78.79 (26.88)	56.67 (29.09)	16.16 (23.74)	21.41 (26.28)	11.11 (19.94)	21.01 (22.76)	35.89 (21.87)
*P* value	**.001**	.941	.289	**<.001**	**<.001**	.117	**.001**	.433	.831	.380	**.001**
Yearly income of family											
>30 000 (304)	60.50 (23.85)	77.85 (21.04)	74.07 (20.97)	73.46 (22.76)	72.81 (29.18)	56.03 (27.59)	20.18 (26.44)	22.26 (27.45)	10.53 (19.50)	19.63 (23.11)	38.78 (22.98)
<30 000 (126)	53.04 (26.93)	70.37 (22.69)	65.81 (26.09)	64.97 (25.03)	68.39 (34.57)	48.68 (32.15)	26.46 (30.79)	23.81 (26.28)	11.64 (20.36)	28.84 (26.45)	45.15 (26.10)
*P* value	**.005**	**.001**	**.001**	**.001**	.177	**.017**	**.033**	.590	.595	**<.001**	**.012**
Interval from symptom onset to diagnosis											
<1 month (72)	77.55 (20.2)	84.95 (16.11)	82.29 (16.84)	83.33 (16.90)	83.10 (23.98)	62.27 (29.87)	14.35 (24.91)	15.28 (24.35)	7.41 (17.89)	8.33 (15.57)	27.47 (20.60)
>1 month (358)	54.45 (24.08)	73.79 (22.31)	69.51 (23.34)	68.49 (24.15)	69.18 (31.61)	52.19 (28.76)	23.56 (28.24)	24.21 (27.40)	11.55 (20.04)	25.14 (24.97)	43.30 (23.88)
*P* value	**<.001**	**<.001**	**<.001**	**<.001**	**<.001**	**.007**	**.010**	**.011**	.105	**<.001**	**<.001**
Distance between the home and the hospital											
Located in the same city (188)	57.00 (26.39)	72.61 (24.04)	71.45 (24.22)	69.22 (25.08)	70.57 (32.10)	55.41 (29.43)	25.00 (29.58)	24.65 (28.03)	12.41 (20.96)	27.84 (26.47)	43.68 (26.10)
Located in a different city (242)	59.33 (23.86)	78.03 (19.58)	71.80 (21.81)	72.34 (22.60)	72.25 (29.94)	52.69 (28.94)	19.70 (26.34)	21.21 (26.30)	9.64 (18.69)	18.04 (21.91)	38.29 (22.15)
*P* value	.339	**.010**	.877	.176	.577	.337	**.050**	.193	.149	**<.001**	**.021**
Psychosocial factors											
Depression											
Yes (191)	46.07 (23.78)	65.27 (23.78)	58.55 (24.22)	61.61 (26.26)	59.25 (34.56)	41.54 (27.88)	32.64 (31.89)	27.92 (28.40)	14.49 (21.76)	30.02 (27.48)	51.25 (25.46)
No (239)	68.10 (21.40)	83.96 (15.75)	82.11 (15.07)	78.47 (18.40)	81.31 (23.41)	63.74 (26.30)	13.53 (20.68)	18.55 (25.29)	7.95 (17.47)	16.18 (19.77)	32.17 (19.11)
*P* value	**<.001**	**<.001**	**<.001**	**<.001**	**<.001**	**<.001**	**<.001**	**<.001**	**.001**	**<.001**	**<.001**
Anxiety											
Yes (204)	47.39 (24.09)	69.44 (21.71)	59.35 (23.41)	63.20 (25.34)	61.52 (33.45)	44.28 (29.64)	29.08 (29.66)	24.35 (27.30)	13.24 (21.03)	28.76 (25.86)	48.26 (24.44)
No (226)	68.18 (21.48)	81.27 (20.32)	82.74 (15.56)	77.99 (19.76)	80.53 (25.23)	62.54 (25.87)	15.63 (24.57)	21.24 (26.87)	8.70 (18.27)	16.52 (21.60)	33.78 (21.60)
*P* value	**<.001**	**<.001**	**<.001**	**<.001**	**<.001**	**<.001**	**<.001**	.235	**.017**	**<.001**	**<.001**
Disease‐ or treatment‐related effects											
Disease phase											
Stable (248)	65.96 (22.34)	78.76 (18.95)	75.57 (20.73)	76.77 (19.45)	79.84 (26.09)	58.94 (28.25)	17.34 (23.80)	20.43 (26.22)	9.54 (19.52)	20.70 (24.40)	35.71 (21.56)
Unstable (182)	47.89 (24.7)	71.43 (24.56)	66.30 (24.57)	63.08 (26.65)	60.16 (33.27)	46.98 (29.03)	28.39 (31.63)	25.82 (28.01)	12.64 (19.95)	24.54 (24.44)	47.37 (25.71)
*P* value	**<.001**	**.001**	**<.001**	**<.001**	**<.001**	**<.001**	**<.001**	**.041**	.108	.108	**<.001**
Treatment‐related toxicities											
≤3 (173)	68.79 (20.89)	83.24 (16.62)	80.68 (18.34)	79.61 (17.85)	82.18 (24.22)	63.68 (25.14)	13.29 (20.57)	17.15 (22.33)	6.74 (15.23)	15.8 (20.82)	30.44 (17.92)
> 3 (126)	57.01 (23.01)	72.75 (20.93)	66.34 (21.46)	68.10 (23.76)	68.65 (31.52)	48.15 (30.57)	28.31 (29.83)	29.89 (31.50)	14.29 (22.48)	26.72 (26.67)	45.15 (23.04)
*P* value	**<.001**	**<.001**	**<.001**	**<.001**	**<.001**	**<.001**	**<.001**	**<.001**	**.001**	**<.001**	**<.001**
Transplantation											
Yes (118)	68.29 (22.49)	79.52 (18.02)	80.01 (17.47)	79.44 (17.12)	83.76 (22.69)	61.16 (26.87)	13.28 (22.70)	17.23 (24.93)	6.21 (15.68)	18.93 (19.72)	31.92 (19.36)
No (124)	60.62 (23.07)	77.02 (19.66)	72.04 (22.09)	71.83 (22.82)	72.31 (31.23)	57.66 (29.25)	22.04 (26.51)	23.92 (28.37)	13.71 (22.89)	22.58 (27.07)	40.32 (23.43)
*P* value	**.009**	.304	**.002**	**.004**	**.001**	.334	**.006**	.053	**.003**	.233	**.003**

	QLQ‐C30 symptom domain scores				QLQ‐MY20				EQ‐5D	
	Insomnia	Nausea and vomiting	Pain	Financial difficulty	Disease symptoms	Side effects of treatment	Body image	Future perspective	EQ‐5D index score	EQ‐5D VAS
Difficulties experienced during the diagnosis and treatment processes										
With caregivers										
Yes (265)	29.94 (29.18)	10.94 (19.51)	34.34 (28.26)	51.82 (34.78)	25.14 (20.39)	28.04 (15.98)	64.53 (28.27)	57.61 (23.81)	0.65 (0.2)	64.19 (21.31)
No (165)	26.06 (30.59)	7.47 (15.44)	24.44 (22.66)	59.19 (34.80)	21.08 (19.30)	26.51 (16.48)	64.04 (28.51)	60.40 (23.52)	0.72 (0.16)	68.29 (21.38)
*P* value	.189	**.042**	**<.001**	**.033**	**.041**	.340	.862	.235	**<.001**	.053
Yearly income of family										
>30 000 (304)	27.96 (28.86)	7.89 (15.07)	28.12 (25.96)	47.37 (32.95)	20.60 (17.23)	25.67 (14.79)	65.35 (28.50)	60.53 (23.38)	0.69 (0.19)	68.86 (20.55)
<30 000 (126)	29.63 (31.89)	13.76 (23.48)	36.38 (27.52)	72.22 (33.40)	30.78 (24.23)	31.75 (18.48)	61.9 (27.88)	54.23 (24.00)	0.65 (0.19)	58.30 (21.66)
*P* value	0.597	**.002**	**.003**	**<.001**	**<.001**	**<.001**	.251	**.012**	**.019**	**< 0.001**
Interval from symptom onset to diagnosis										
<1 month (72)	23.61 (24.67)	5.32 (15.53)	15.05 (19.81)	42.13 (37.53)	12.58 (11.87)	20.23 (13.81)	69.44 (25.48)	65.74 (22.97)	0.78 (0.09)	78.32 (15.35)
>1 month (358)	29.42 (30.61)	10.47 (18.49)	33.66 (26.80)	57.17 (33.89)	25.79 (20.64)	28.90 (16.24)	63.31 (28.79)	57.26 (23.63)	0.66 (0.20)	63.24 (21.58)
*P* value	0.131	**0.028**	**<.001**	**.001**	**<.001**	**<.001**	.094	**.005**	**<.001**	**<.001**
Distance between the home and the hospital										
Located in the same city (188)	30.32 (30.2)	11.35 (19.46)	30.5 (27.27)	52.13 (34.84)	25.03 (19.92)	29.91 (17.08)	62.06 (28.88)	59.63 (25.59)	0.67 (0.19)	64.73 (23.23)
Located in a different city (242)	27.00 (29.38)	8.26 (16.92)	30.58 (26.24)	56.61 (34.95)	22.45 (20.13)	25.54 (15.20)	66.12 (27.83)	57.94 (22.16)	0.68 (0.19)	66.57 (19.89)
*P* value	.251	.08	.975	.187	**.186**	**.005**	.141	.464	.433	.378
Psychosocial factors										
Depression										
Yes (191)	38.22 (32.97)	15.79 (22.99)	39.88 (28.34)	63.70 (32.76)	30.16 (21.68)	33.58 (17.12)	53.75 (30.52)	46.66 (23.92)	0.59 (0.22)	54.99 (21.63)
No (239)	20.64 (24.29)	4.67 (10.71)	23.08 (22.68)	47.42 (34.99)	18.32 (16.94)	22.55 (13.54)	72.80 (23.26)	68.29 (18.64)	0.75 (0.12)	74.37 (16.87)
*P* value	**<.001**	**<.001**	**<.001**	**<.001**	**<.001**	**<.001**	**<.001**	**<.001**	**<.001**	**<.001**
Anxiety										
Yes (204)	36.60 (31.91)	13.56 (20.49)	40.52 (28.37)	62.91 (32.11)	29.52 (21.00)	32.94 (17.03)	57.84 (30.30)	46.84 (23.25)	0.61 (0.21)	57.48 (21.49)
No (226)	21.09 (25.58)	6.05 (14.83)	21.53 (21.39)	47.20 (35.76)	18.22 (17.54)	22.49 (13.61)	70.21 (25.09)	69.37 (18.50)	0.74 (0.14)	73.24 (18.40)
*P* value	**<.001**	**<.001**	**<.001**	**<.001**	**<.001**	**<.001**	**<.001**	**<.001**	**<.001**	**<.001**
Disease‐ or treatment‐related effects										
Disease phase										
Stable (248)	25.13 (27.82)	6.79 (14.77)	23.32 (21.51)	50.67 (35.48)	19.76 (18.21)	26.45 (15.09)	64.92 (25.66)	62.77 (21.77)	0.73 (0.14)	71.50 (19.47)
Unstable (182)	32.97 (31.73)	13.46 (21.32)	40.38 (29.74)	60.07 (33.51)	28.79 (21.29)	28.81 (17.50)	63.55 (31.67)	53.11 (25.12)	0.61 (0.22)	57.95 (21.50)
*P* value	**.007**	**<.001**	**<.001**	**.006**	**<.001**	.135	.622	**<.001**	**<.001**	**<.001**
Treatment‐related toxicities										
≤3 (173)	21.77 (26.56)	3.85 (9.23)	20.71 (20.25)	46.82 (33.50)	15.67 (14.29)	21.48 (13.07)	70.13 (22.75)	66.15 (21.19)	0.74 (0.14)	72.51 (18.41)
> 3 (126)	33.60 (29.06)	12.83 (21.52)	32.01 (24.73)	61.64 (35.29)	26.37 (22.10)	32.06 (16.04)	58.73 (29.64)	53.97 (22.91)	0.66 (0.19)	66.70 (20.34)
*P* value	**<.001**	**<.001**	**<.001**	**<.001**	**<.001**	**<.001**	**<.001**	**<.001**	**<.001**	**.01**
Transplantation										
Yes (118)	24.58 (29.70)	4.52 (11.04)	19.77 (19.85)	46.61 (34.63)	17.56 (15.18)	25.34 (14.93)	63.84 (26.36)	66.85 (22.03)	0.75 (0.12)	73.03 (20.11)
No (124)	27.42 (29.16)	8.87 (17.96)	27.69 (25.76)	54.30 (36.68)	21.42 (21.58)	26.02 (15.40)	68.01 (25.99)	58.15 (22.67)	0.69 (0.18)	67.32 (18.94)
*P* value	.453	**.025**	**.008**	.095	.111	.727	.217	**.003**	**.002**	**.024**

Bold values denote significant *P* values (<.05).

### 3.3 Psychosocial factors associated with HRQOL

According to the HADS, 44.4% (191) of patients scored 8 or more on the anxiety scale, and 47.4% (204) scored 8 or more on the depression scale. Both depression and anxiety were associated with a general trend toward worse EORTC QLQ‐C30 plus QLQ‐MY20 scores and EQ‐5D index (Table 3).

### 3.4 Disease‐ or treatment‐related effects associated with HRQOL

A total of 248 (57.7%) patients were currently in the stable disease phase of MM. The component scores for the functioning and symptom scores of QLQ‐C30 plus QLQ‐MY20 and EQ‐5D scores were significantly higher in the stable disease phase than in the unstable disease phase. Two hundred and forty‐two patients reported an ASCT eligibility status, including 118 who underwent ASCT and 124 who were not treated with ASCT. The component scores of QLQ‐C30 plus QLQ‐MY20 and EQ‐5D were significantly better in the patients with MM who underwent ASCT (Table 3).

**Figure 1:**
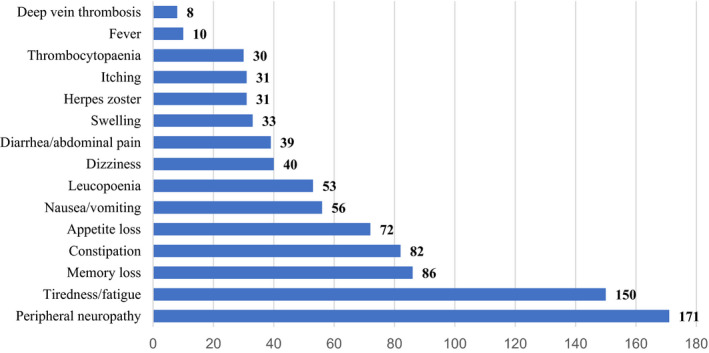
Treatment‐related toxicities

**Figure 2:**
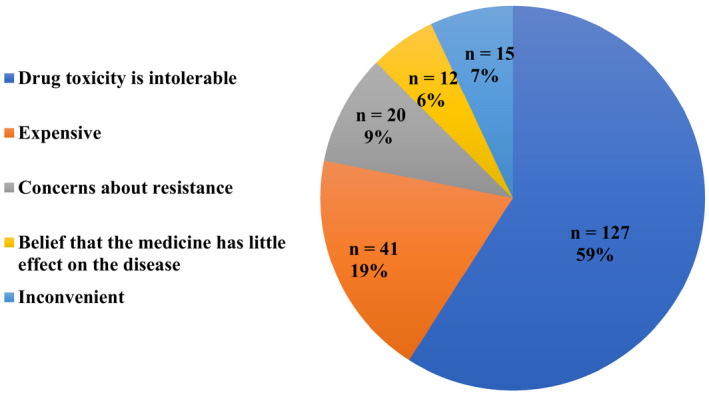
Reasons for stopping maintenance treatment

A total of 299 patients received MM treatment and reported treatment‐related toxicities. The treatment‐related toxicities were indicated in this survey (Figure 1). The patients had an average of 3.16 treatment‐related toxicities (SD 1.65, range 0‐6); patients with fewer treatment‐related toxicities reported an improved HRQOL across all domains of the QLQ‐C30 plus QLQ‐MY20 and EQ‐5D index (Table 3). Furthermore, most of the 235 patients (n = 215, 91.5%) indicated that they prefer to discontinue MM maintenance treatment in the future. The predominant reason for preferring to stop treatment was treatment‐related adverse events (n = 127, 59%), the high cost (n = 41, 19%), concerns about treatment resistance (n = 20, 9%), inconvenience in daily life (n = 15, 7%), and the belief that taking medicine has little effect on the disease (n = 12, 6%) (Figure 2).

### 3.5 Multivariate regression analysis of HRQOL

In the multivariate linear regression model, the quality of life score (QLQ‐C30 global health status and EQ‐5D index) was significantly and independently associated with the performance status (ECOG > 2), higher levels of anxiety and depression, an unstable disease phase of MM, and a late diagnosis of MM (Table 4).

**Table 4:** Multivariate linear regression models of the EORTC QLQ‐C30 global health status and EQ‐5D index

	*P* value	QLQ‐C30 global health status			*P* value	EQ‐5D index		
		Coefficient	Lower CI	Upper CI		Coefficient	Lower CI	Upper CI
Gender	.405	−1.651	−5.545	2.242	.611	0.008	−0.022	0.037
Age	.425	−2.081	−7.203	3.042	.301	−0.021	−0.060	0.018
Working status	.689	−0.998	−5.902	3.901	.949	−0.001	−0.039	0.036
Education level	.067	4.907	−0.352	10.166	.610	0.010	−0.030	0.051
ECOG 2‐4 degrees	**<.001**	−9.661	−13.901	−5.421	**<.001**	−0.153	−0.185	−0.121
Yearly income	.573	1.330	−3.299	5.958	.764	0.005	−0.030	0.041
Caregiver	.266	2.248	−1.723	6.219	.163	0.022	−0.009	0.052
Timely diagnosis	**<.001**	−15.435	−20.531	−10.339	**.010**	−0.067	−0.106	−0.028
HADS anxiety	**<.001**	9.124	4.731	13.517	**.004**	0.049	0.015	0.082
HADS depression	**<.001**	10.648	6.159	15.136	**<.001**	0.067	0.033	0.101
Unstable disease phase	**<.001**	−10.939	−14.791	−7.086	**<.001**	−0.061	−0.090	−0.031
R2	.424				.418			

Bold values denote significant *P* values (<.05).

## 4 DISCUSSION

MM is a common hematological cancer. Despite significant improvements in its treatment, MM remains a chronic incurable disease associated with a reduced HRQOL due to bone involvement and fractures, recurrent bacterial infections, impaired renal function, anemia, mood disorders accompanied by reduced physical functioning, and side effects of the different types of treatments used to control this disease. The findings of a previous study showed that patients with MM experience a much lower HRQOL than the general population regardless of the number of years since diagnosis.^6^ Evidence suggests that myeloma patients suffer from more symptoms and problems than patients with other hematological cancers. A study conducted in Denmark reported a mean symptom level of 5.6 symptoms with 2.3 symptoms identified as severe. MM patients reported the highest level of pain, fatigue, and constipation along with problems in physical, role, and social functioning. Additionally, previous studies have demonstrated that the HRQOL of patients with MM had the lowest mean rank score compared with patients with other hematological cancers.^18^, ^19^, ^20^

Therefore, the quality of life of patients must be improved.^4^ Thus, the focus of attention has shifted to obtaining the most durable remissions with the highest HRQOL. Increasingly, HRQOL analyses are included in clinical trials to assess how the HRQOL is affected by a course of treatment. According to previous studies, the extent of HRQOL impairment in MM may vary depending on the burden of MM‐related symptoms, treatment‐related toxicities, patient‐related factors, and psychosocial factors.^21^ Additionally, the quality of life may vary according to the cultural and health service contexts in different countries.

However, to the best of our knowledge, few reports have comprehensively described the HRQOL of patients with MM in China. In Chinese public hospitals, the situation of MM patients completely differs from that in other countries; thus, we designed a questionnaire based on the existing literature and the characteristics of China to evaluate the HRQOL in Chinese MM patients and suggest how to improve their HRQOL. This article aims to apply the QLQ‐C30, QLQ‐MY20, and EQ‐5D to evaluate the HRQOL of 430 patients with different stages of MM, analyze the factors affecting the quality of life, and specify the best strategy for improving the quality of life of patients with MM in China.

### 4.1 Patient factors

Based on the findings from the present study, women had a lower HRQOL than men in multiple functional and symptom domains, consistent with the findings from studies of other cancer types.^22^ Therefore, medical staff and family caregivers should pay attention to the quality of life of female patients. MM is a disease affecting older people, and studies investigating MM have demonstrated that an older age predicts an overall worse HRQOL.^18^, ^23^ As expected, our findings demonstrate that the global health status, physical functioning, and symptom domain all tended to decline with advancing age. In the univariate analysis, age and gender appeared to be key factors associated with patient‐reported HRQOL in MM. However, in the multiple linear regression analysis, age and gender were not independent prognostic factors affecting the global HRQOL of MM patients. Other researchers have also reported conflicting results when evaluating the HRQOL in MM. Age did not predict the EORTC QLQ‐C30 global health status in a smaller European cohort study involving a cross‐sectional analysis of all patients presenting MM regardless of the disease or treatment stage. The reason may be that a lower performance level, increased comorbidities, and the treatment stage of the older population may play a larger role than the biological age.^24^, ^25^

In addition to age and gender, various other demographic and baseline clinical variables significantly impact the HRQOL. According to Priscilla et al^26^ employed patients with cancer experience a higher HRQOL than patients who are unemployed, which is consistent with our results of patients with MM. Thus, interventions targeted at patients with MM should focus on their ability to return to work. In the present study, the HRQOL of urban patients was higher than rural patients, and a higher education level predicts a better overall HRQOL. Rural patients had little disease‐related knowledge and lower education level; therefore, more health education should be provided to patients in rural areas to improve their quality of life.

Notably, Robinson et al^27^ reported an association between a worse ECOG and a lower HRQOL. In our study, the quality of life of patients with ECOG grade 2 or higher was poor. The ECOG was identified as a factor independently associated with the global HRQOL in the multivariate linear regressions; thus, an estimation of performance status is very important.

### 4.2 Difficulties experienced during the diagnosis and treatment processes

Due to receiving treatment for the disease, the patient's ability to take care of themselves decreases, and the caregiver is required to provide daily care.^28^ In our study, 165 (38.4%) patients with MM had no caregiver during treatment. The absence of caregivers is the current status of patients with MM in China. Access to support from family and friends or health‐care professionals has been shown to be beneficial to the HRQOL of patients with MM.^29^ The improved HRQOL scores may have been due to the patients having better access to supportive care. Supportive care and caregivers are essential for myeloma patients because while directed toward improving the patient's quality of life, they also have significant effects against the disease and can improve survival. Furthermore, caregivers can help patients with MM receive more material and spiritual help. They can also enhance patients’ confidence in overcoming the disease and reduce the anxiety and depression of patients, thus improving their HRQOL.^28^, ^29^

Economic difficulties and unfavorable factors are commonly experienced during the treatment of various types of cancer. MM treatment requires not only expensive chemotherapy drugs but also additional supportive treatment, which will lead to an increase in the economic burden on patients.^30^ Based on the results from the present study, a lower yearly family income (<30 000 RMB) resulted in a lower HRQOL of patients with MM.

MM is difficult to diagnose at early stages and is often misdiagnosed as other diseases; thus, the opportunity for timely treatment is also lost. Only 72 (16.7%) patients were diagnosed with MM within 1 month after the onset of the first symptoms. Further investigations of the reasons for the patient's failure to receive an early diagnosis of myeloma are necessary. The reasons included: a lack of awareness of the severity of the disease (n = 167, 46%), the hospital failing to confirm the diagnosis in a timely manner (n = 159, 43%), and the inability of the patient to continue due to economic reasons (n = 41, 11%). The early diagnosis of MM improved the patients’ HRQOL and was an independent influencing factor in multivariate linear regressions, highlighting the importance of an early diagnosis. No relevant reports have described these results. Therefore, an improved awareness of MM in doctors and patients is conducive to improving the HRQOL of patients.

In China, due to the uneven distribution of medical resources, many patients with MM must travel to other large‐sized cities to seek medical treatment. A total of 242 (56.3%) patients in our study stated that the hospital was not located in the same city as their home. The long distance between the home and hospital increases the difficulties experienced during diagnosis and treatment processes. In terms of HRQOL, the location of the hospital and home in different cities resulted in worse scores on some functional and symptom subscales but had little effect on the overall HRQOL, which may be related to the development of the transportation system in China. However, the relationship between the locations of the home and the hospital is an undesirable factor that medical workers must consider.

### 4.3 Psychosocial factors

Living with a diagnosis of MM has been consistently shown to impact HRQOL.^31^ Psychosocial issues were present in up to 40% of patients with MM in our study and have been described in previous reports^18^; anxiety and depression were significant independent factors associated with all outcomes. The importance and persistence of mental health problems as predictors of survival have been reported in other studies.^32^, ^33^ Patients live with the uncertainly of a treatable but incurable cancer; they worry about how their illness will progress and are concerned about death and dying.^28^ Overall, these findings suggest that patients need more psychological support during all phases of treatment.

### 4.4 Disease‐ and treatment‐related effects

The HRQOL for patients diagnosed with MM is poor compared to that of patients living with advanced cancer.^26^, ^34^ Disease‐ and treatment‐related effects adversely affect all domains of HRQOL. Patients with MM generally experience the highest level of symptoms and lower HRQOL at diagnosis, during periods of active treatment, and when experiencing progressive disease. Patients experience fewer symptoms and the highest levels of HRQOL during their treatment‐free intervals.^6^, ^35^ The HRQOL of patients with RRMM is influenced by their disease‐related symptoms, treatment‐related toxicity, and treatment response. Long‐term survivors of advanced MM report that the cumulative impact of intensive treatment can significantly impact the HRQOL mainly due to the burden of disease‐related and treatment‐related symptoms.^36^ Indeed, a European, multicenter cohort study found that MM disease symptoms were associated with significant reductions in the HRQOL. Receiving any type of MM treatment was linked to significant reductions in the HRQOL likely due to treatment‐related side effects.^7^ Our results also confirmed this finding. Treatment during the unstable phase can have an adverse impact on the HRQOL and is an independent factor according to the multivariate linear regressions. Therefore, relieving MM disease through treatment effectively improves HRQOL.

The application of ASCT has significantly improved the prognosis of patients with MM and prolonged the OS of patients.^37^, ^38^ However, as patient survival is prolonged, patients experience different levels of pain due to treatment‐related toxicities. People who received ASCT began to pay attention to the HRQOL. As shown in the study by Etto et al,^39^ ASCT improves the quality of life in Brazilian patients with MM. However, some studies have shown that ASCT can have a transient adverse impact on HRQOL.^40^ In our study, 118 patients underwent ASCT, and 124 patients did not accept ASCT. Furthermore, we investigated why patients did not undergo ASCT, including economic reasons (n = 45, 36%), an unsuitable physical status for ASCT (n = 40, 32%), fear of ASCT (n = 20, 16%), stem cell collection failure (n = 8, 7%), and lack of doctor recommendation for ASCT (n = 5, 4%) or hospital lacking facilities suitable for ASCT (n = 6, 5%). In our study, patients showed a significant improvement in overall HRQOL after ASCT compared with those who did not undergo ASCT, indicating a positive effect on the overall disease outcome. Therefore, ASCT is the recommended treatment.

Indeed, a European, multicenter cohort study reported that the receipt of any type of MM treatment was linked to significant reductions in the HRQOL likely due to treatment‐related side effects.^7^, ^36^ In our study, patients reported a wide range of treatment‐related toxicities; the most commonly experienced and most bothersome was peripheral neuropathy. Studies consistently find that treatment‐related toxicities are predictors of HRQOL. Our study reported a mean of 3.16 symptoms. A higher prevalence of treatment‐related toxicities is experienced by patients with MM.^41^, ^42^ Patients with MM who experienced more than three symptoms reported a poorer HRQOL. Thus, we considered the impact of long‐term maintenance therapy on patients’ HRQOL. Notably, 91.5% of patients were willing to discontinue maintenance therapy. The predominant reason for preferring to stop treatment was treatment‐related toxicities, the high cost, concerns about treatment resistance, inconvenience in daily life, and the belief that taking medicine has little effect on the disease. Adverse events related to maintenance therapy were an important reason why patients might prefer to stop therapy in the future. Thus, the period of maintenance treatment is a problem we must solve in the future.

A limitation of our study is that this study employed a cross‐sectional design. Therefore, the independent variables included in the regression analyses and any correlations reported represent associations but not predictions. Physiological variables indicating disease activity were not extracted from the medical records and not considered in the regression analysis. Nevertheless, our study is noteworthy because we believe that this study is the first real‐world study in China to evaluate the HRQOL of patients with MM, and the results can increase our understanding of priority problems in this population. Furthermore, recognizing the factors influencing the HRQOL can help us better target health outcomes, better understand individual patients’ needs and impact of interventions, and plan patient treatment regimens and supportive care, which have the potential to further improve the HRQOL of patients living with MM. In the future, we need to further study the impact of the quality of life on the prognosis of patients and conduct longitudinal studies and intervention studies investigating the HRQOL of MM patients.

## 5 CONCLUSIONS

Patient factors, difficulties experienced during the diagnosis and treatment processes, psychosocial factors, and disease‐ or treatment‐related effects are significantly associated with the HRQOL of patients with MM in China, particularly for the patients with a low performance status (ECOG > 2), higher levels of anxiety and depression, an unstable disease phase of MM, and a lack of an early diagnosis of MM. Efforts should be made to identify persons at risk of low HRQOL earlier and improve the overall quality of life of these patients in China.

## INFORMED CONSENT

Informed consent was obtained from all individual participants included in the study.

## CONFLICT OF INTEREST

The authors declare that they have no conflict of interest to disclose.

## AUTHOR CONTRIBUTIONS

All authors contributed to the study conception and design. Material preparation, data collection, and analysis were performed by Junru Liu, Meilan Chen, Jingli Gu, Beihui Huang, and Dong Zheng. The first draft of the manuscript was written by Juan Li and Xiaozhe Li and all authors commented on previous versions of the manuscript. All authors read and approved the final manuscript.

## ETHICAL APPROVAL

All procedures performed in studies involving human participants were conducted in accordance with the ethical standards of the institutional and/or national research committee and with the 1964 Declaration of Helsinki and its later amendments or comparable ethical standards.

## Data Availability

The authors confirm that the data supporting the findings of this study are available within the article and its supplementary materials.
